# Emerging role and function of SPDL1 in human health and diseases

**DOI:** 10.1515/med-2024-0922

**Published:** 2024-04-07

**Authors:** Yuejiao Feng, Donghao Tang, Jie Wang

**Affiliations:** Shanghai Putuo Central School of Clinical Medicine, Anhui Medical University, Shanghai, 200062, China; The Fifth School of Clinical Medicine, Anhui Medical University, Anhui, 230022, China; Department of General Surgery, Putuo Hospital, Shanghai University of Traditional Chinese Medicine, Shanghai, 200062, China

**Keywords:** cancer, CCDC99, cell cycle, hSpindly, *SPDL1*

## Abstract

*SPDL1* (spindle apparatus coiled-coil protein 1), also referred to as CCDC99, is a recently identified gene involved in cell cycle regulation. *SPDL1* encodes a protein, hSpindly, which plays a critical role in the maintenance of spindle checkpoint silencing during mitosis. hSpindly coordinates microtubule attachment by promoting kinesin recruitment and mitotic checkpoint signaling. Moreover, the protein performs numerous biological functions *in vivo* and its aberrant expression is closely associated with abnormal neuronal development, pulmonary interstitial fibrosis, and malignant tumor development. In this review, we provide an overview of studies that reveal the characteristics of *SPDL1* and of the protein encoded by it, as well as its biological and tumor-promoting functions.

## 1 Introduction

Biomedical research has greatly improved the understanding of complicated cancers. Aberrant proliferation and cell cycle signals are the major hallmarks of cancer [1,2]. Many genes have been found to be dysregulated in cancer cells and participate in tumor progression [3]. *SPDL1* (spindle apparatus coiled-coil protein 1), also referred to as *CCDC99*, is a recently identified gene that has been shown to be involved in cell cycle regulation [4]. *SPDL1* encodes a protein named hSpindly that is involved in the segregation of chromosomes during the cell cycle, for example, during spindle formation in mitosis and the spindle assembly checkpoint (SAC). The mitotic checkpoint complex (MCC) is a single stable complex, consisting of the proteins hBUBR1, hBUB3, CDC20, and MAD2 [5]. After the MCC formation, silent SAC makes abnormal mitotic chromosomes during the period of separation, leading to the formation of aneuploid. This is associated with tumor formation and diseases, such as spontaneous abortion and congenital birth defects [6–9]. Aberrant expression of *SPDL1* is strongly associated with the development of multiple tumors [10]. hSpindly also interacts with Rod/ZW10/Zwilch (RZZ) and dynein–dynactin complexes and plays a vital role in cell migration [11–19]. In this review, we summarize the molecular structure and biological functions of hSpindly and its function in various tumors.

## 2 Gene and protein structure of hSpindly

**Figure 1:**
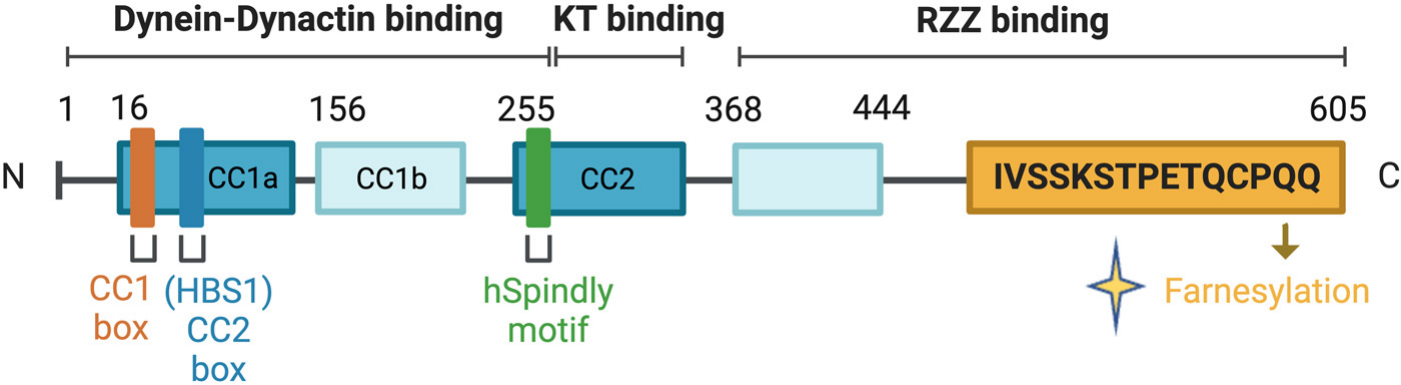
Domain structure of the human hSpindly proteins. hSpindly undergoes farnesylation *in vivo* at a cysteine residue in its C-terminal domain. The N-terminal residues 293–322 contribute to the high-affinity binding with KT of hSpindly. Created with BioRender.com.

hSpindly was originally identified as a protein in a screen in *Drosophila melanogaster* S2 cells and it can cause a mitotic arrest and changes in cell morphology. Drosophila (Dm) Spindly is an 807-residue protein characterized by the presence of predicted coil-rich regions in its N-terminal half. The human *SPDL1* gene is located on chromosome 5q35.1 [20,21]. The protein encoded by this gene comprises 605 amino acids and has a molecular weight of 70,172 Da. The protein ID of Spindly was Q96EA4 [22]. To distinguishing between the Drosophila and human SPDL1 gene, the human *SPDL1* gene will be referred to as “hspindly” hereafter. hSpindly has a transcript length of 2,529 bps, associated with 6,057 variant alleles and maps to 467 oligo probes. This transcript has 12 exons, 11 coding exons, is annotated with 20 domains and features. The protein contains a coiled helix structural domain that comprises two coiled domains with a conserved 32-amino acid spindle sequence in the middle [23]. The structure of hSpindly also includes the following four repetitive motifs: 553 1P-KPQLKGTPVK 564, 591 TPAKPLMKGTPVK 603, 627 TPAKPQRKGTPVK 639, and 675 TPQKPQRKGTPVR 687, with a consensus sequence of TPXKPQXKGTPVK [18]. hSpindly undergoes farnesylation *in vivo* at a cysteine residue in its C-terminal domain. The N-terminal residues 293–322 contribute to the high-affinity binding with KT of hSpindly [23] (Figure 1).

hSpindly localizes to the nucleus in interphase and to the kinetochore (KT) in early prometaphase. It relocalizes to the mitotic spindle pole before metaphase and is subsequently lost from the spindle poles after chromosome congression is completed [22]. Removal of hSpindly from the KT requires the dynein/dynactin complex. Moreover, depletion of hSpindly and ZW10 in turn affects the subcellular localization of dynamin [16].

## 3 Biological functions of hSpindly

### 3.1 Regulation of cell mitosis by hSpindly

hSpindly facilitates microtubule (MT) attachment and is involved in MCC silencing. It participates in SAC silencing, and thereby, in the inhibition of MCC formation. hSpindly regulates SAC and can remove external KT components and SAC proteins more quickly by recruiting the MT kinesin, dynein to compress KTs [24,25]. The removal of SAC proteins from KTs requires the dynein–dynactin complex, and hSpindly is required to target dynein to KTs [20,26]. In addition, hSpindly localizes to the nucleus during interphase and to KT during prophase; it relocates to the mitotic spindle pole until the metaphase–anaphase transition and is subsequently removed from the spindle pole upon completion of chromosome convergence (congression) [27]. *SPDL1* knockdown causes unstable interactions between mitotic granules and MTs, leading to prolonged mitosis [28].

The segregation of chromosomes is one of the most critical processes for the even distribution of genetic material between two daughter cells during mitosis. In all eukaryotic cells, segregation of chromosomes is driven by the spindle [29]. Additionally, it requires the stable bipolar attachment of spindle MTs to KTs, which is stabilized by the tension generated by KTs, making targeted connections to the opposite poles [30,31]. hSpindly is a KT-specific junction of cytoplasmic kinesin that is important for chromosome pairing. It participates in the regulation of SAC function, and its inhibition can silence SAC by recruiting the dynein–dynactin complex to target KTs, resulting in extensive aggregation of chromosomes and failure to properly segregate them [27,32,33]. The SAC signaling pathway is crucial in regulating the precise segregation of chromosomes during mitosis, ensures that all chromosomes are aligned at the midpalate and that all sister chromatids segregate to the ends of the spindle and are evenly distributed to daughter cells [9,20,34–36].

### 3.2 hSpindly is involved in protein modification *in vivo*

hSpindly was recently shown to regulate protein activity and function through ubiquitination, a protein modification process that involves multiple cellular processes [37]. In cells, the activity of proteins can be regulated by post-translational modifications; these modifications are essential for protein regulation and degradation. Conte identified hSpindly as a new target of the ubiquitin-specific protease USP45; hSpindly and USP45 can form a complex whose interaction is specifically related to the catalytic activity of USP45 [38].

Ubiquitination is a reversible process. hSpindly is monoubiquitinated; the ubiquitin molecule can be specifically removed by the active form of USP45 rather than by the inactive catalytic form. Potential lysine ubiquitination sites of hSpindly were identified using mass spectrometry to be present in the first coiled-coil structural domain. Nevertheless, to date, the exact ubiquitin-binding site of hSpindly has not been identified.

### 3.3 Farnesylation of hSpindly

Scores of proteins are farnesylated in cells. The nonpolar farnesyl group increases the possibility of binding and anchoring farnesylated proteins to the cell membrane. For example, rat sarcoma (Ras) proteins require post-translational farnesylation to bind to the cell membrane and perform signaling functions. The interaction of hSpindly with the RZZ complex *in vivo* is greatly dependent on its farnesylation [39]. Cells treated with farnesyltransferase inhibitor or *SPDL1* knockdown exhibit the same mitotic phenotype, indicating the importance of hSpindly farnesylation.

**Figure 2:**
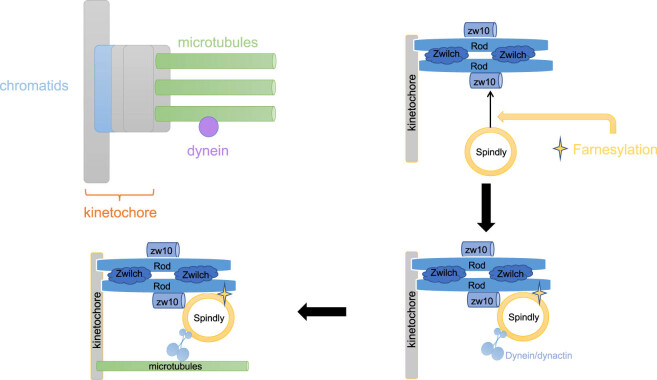
hSpindly is farnesylated *in vivo*. Its interaction with the RZZ complex depends heavily on farnesylation. In addition, the localization of KTs is critical. Created with BioRender.com.

hSpindly was reported to be farnesylated at a cysteine residue in the structural C-terminal domain. The N-terminal 293 amino acids are not necessary for targeting the protein to KTs, and residues 293–322 are involved in high-affinity binding to KT [23]. The deletion of residues in this region may propel premature interaction between the hSpindly N4 structure and the kinesin complex, which translocates the structure from the KT to the spindle pole. Arguably, farnesylation targets hSpindly to the KT (Figure 2).

## 4 hSpindly is associated with a variety of pathological activities *in vivo*

**Figure 3:**
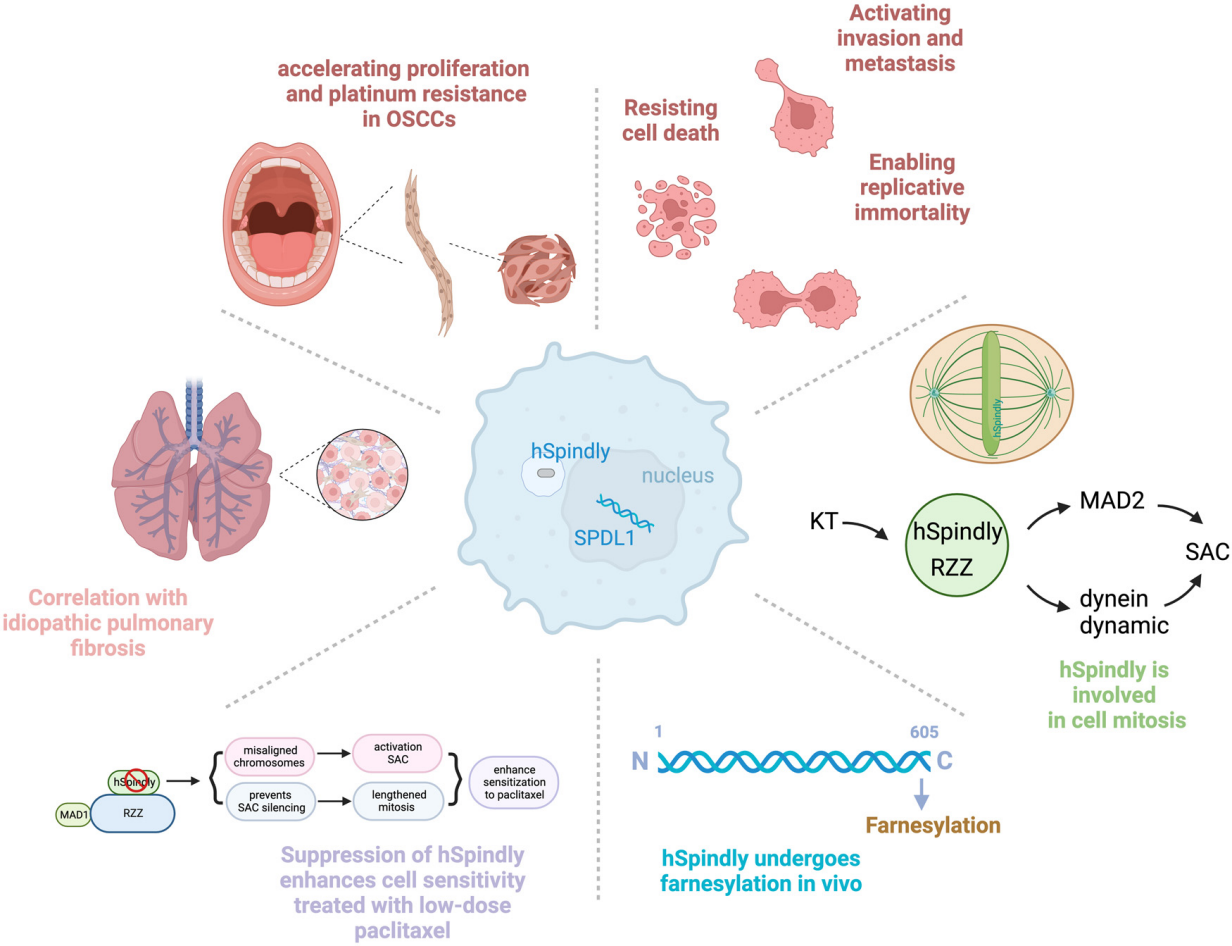
The function of hSpindly. hSpindly is related to various pathological activities *in vivo*, such as protein modification, regulation of neural polarity in Drosophila, participation in the cell cycle and promotion of the development of certain tumors. Created with BioRender.com.

hSpindly exhibits a variety of functions *in vivo*; it is involved in cell cycle, mitosis, and cycle checkpoints (Figure 3). These processes are related to genome composition and cell proliferation, indicating that abnormal expression of *SPDL1* may cause genomic instability (GIN), which may lead to disease development and tumor progression [40].

### 4.1 High SPDL1 expression is positively associated with GIN

GIN is critical in cancer development. It is an abnormal cellular state related to increased rates of inherited genomic alterations (such as mutations and chromosomal rearrangements, deletions, and inversions) and promotes tumorigenesis by increasing the mutation rate of oncogenes during the multistep progression of cancer [41]. GIN is attributed to multiple factors, among which chromosome instability (CIN) and microsatellite instability (MIN) have received the most attention [42–45]. The association between the MIN phenotype and cancer is well established; however, the role of CIN in cancer remains unclear. Studies have focused on the molecular basis of CIN, including its role in spindle checkpoint defects and other mitotic regulators. Studies have shown that the persistent nonadhesion of MTs to chromosomes is the main mechanism of CIN [46].

Tumor cells progress from a less malignant phenotype to a more malignant phenotype owing to inherent instability within the genome. It has also been proposed that the rate of both GIN and metastatic variant production increases as tumor cells reach a higher malignant state [47]. Recent studies on pancreatic ductal carcinoma (PDAC) and colorectal cancer (CRC) suggested that high *SPDL1* expression is associated with GIN [10].

*SPDL1* is considered to be responsible for GIN in PDAC [48]. The main drivers of tumorigenesis related to the prognosis of different cancer types include DNA replication, damage repair, and chromosomal segregation. Defects in these three closely linked processes in the cell cycle, which are also the major sources of GIN, are related to tumor metastasis [49]. hSpindly participates in mitosis in tumors and in the chromosome segregation process, impelling GIN.

The expression of *SPDL1* is also believed to be correlated with the expression of forkhead box protein M1 (Foxm1), a significant predictor of GIN [50]. The overexpression of *SPDL1* in tumor tissues was found to be related to improved survival, CIN phenotype, and different types of GIN markers [40]. As revealed by an integrative analysis of research, the expression of *SPDL1* in CRC was significantly associated with GIN phenotypes (including the CIN/aneuploidy score) and was related to the expression of DNA break repair (MMR) genes, affecting the proliferative activity of tumor cells.

### 4.2 Misaligned variants of SPDL1 increase the risk of pulmonary fibrosis

The function of *SPDL1* in the normal cell cycle is related to the regulation of cycle activity, and hence, the expression of mutated *SPDL1*, such as those with missense mutations, can result in an increased risk of some diseases (Table 1).

**Table 1:** Missense mutations in SPDL1 associated with human diseases

Sample ID	Cancer type	Protein change	Start Pos	End Pos	Ref	Var
TCGA-AR-A24Q-01	Breast cancer	RANBP17-SPDL1 Fusion	170881896			
TCGA-43-5668-01	Non-small cell lung cancer	Q316H	169023621	169023621	G	T
TCGA-90-A59Q-01	Non-small cell lung cancer	EasaK	169025996	169025996	G	A
TCGA-NC-A5HF-01	Non-small cell lung cancer	D541H	169028580	169028580	G	C
TCGA \[-37-4130-01]\]	Non-small cell lung cancer	S54G	169018052	169018052	A	G
TCGA-DK-A686-01	Bladder cancer	Q271.	169021605	169021605	C	T
TCGA-GD-A76B-01	Bladder cancer	Q483E	169028406	169028406	C	G
TCGA-K4-A5RJ-01	Bladder cancer	X442_plice	169028281	169028281	C	\[{\mathrm{G}}]\]
TCGA-K4-A5RJ-01	Bladder cancer	\[5515{\mathrm{C}}]\]	169028503	169028503	C	G
TCGA-ED-A459-01	Hepatobiliary cancer	T3111	169023605	169023605	C	T
TCGA-DD-AAEB-01	Hepatobiliary cancer	R203L	169021225	169021225	G	\[{\mathrm{T}}]\]
TCGA-FC-7961-01	Prostate cancer	N391	169015536	169015536	A	T
TCGA-XIK-AAIW-01	Prostate cancer	S575L	169031117	169031117	C	T
TCGA-CZ-5456-01	Renal clear cell carcinoma	K437N	169026150	169026150	A	C
TCGA-B2-5633-01	Renal clear cell carcinoma	D459E	169028336	169028336	\[{\mathrm{T}}]\]	A
TCGA-A5-AOG2-01	Endometrial cancer	\[{\mathrm{R}}479{\mathrm{Q}}]\]	169028395	169028395	G	A
TCGA-D1-A17Q-01	Endometrial cancer	M48I	169015564	169015564	G	T
TCGA-B5-A0JY-01	Endometrial cancer	D465N	169028352	169028352	G	A
TCGA-A5-AOVQ- 01	Endometrial cancer	R264Q	169021595	169021585	G	A
TCGA-AP-A051-01	Endometrial cancer	X442 splice	169026165	169026165	\[{\mathrm{T}}]\]	\[{\mathrm{G}}]\]
TCGA-AX-A010-01	Endometrial cancer	D432E	169026135	169026135	T	\[{\mathrm{G}}]\]
TCGA-64-5781-01	Non-small cell lung cancer	\[{\mathrm{V}}522\hspace{1em}{\mathrm{L}}]\]	169028523	169028523	G	\[{\mathrm{T}}]\]
TCGA-64-5781-01	Non-small cell lung cancer	\[{\mathrm{A}}542{\mathrm{P}}]\]	169028593	169028583	G	C
TCGA-55-7576-01	Non-small cell lung cancer	M12	169015423	169015423	G	\[{\mathrm{T}}]\]
TCGA-05-4382-01	Non-small cell lung cancer	A3P	169015427	169015427	G	C
TCGA-50-5072-01	Non-small cell lung cancer	Q310Nfz-2	169023598	169023599	TC	—
TCGA-50-5930-01	Non-small cell lung cancer	\[{\mathrm{R}}45{\mathrm{H}}]\]	169015554	169015554	G	A
TCGA-BR-A-4J8-01	Esophagogastric cancer	\[{\mathrm{R}}230{\mathrm{H}}]\]	169021405	169021405	G	A
TCGA-HU-A.4GX-01	Esophagogastric cancer	K.494Rfz*10	169028407	169028410	AGAA	—
TCGA-BR-AAJJ5-01	Esophagogastric cancer	E183del	169021159	169021161	AAG	—
TCGA-CG-5733-01	Esophagogastric cancer	E183del	169021159	169021161	AAG	—
TCGA-BR-4280-01	Esophagogastric cancer	\[{\mathrm{Y}}591{\mathrm{H}}]\]	169031164	169031164	T	C
TCGAVQ-A92D-01	Esophagogastric cancer	T111lfs*3	169018219	169018219	A	—
TCGA-CG-4442-01	Esophagogastric cancer	\[{\mathrm{H}}332{\mathrm{R}}]\]	169023668	169023668	A	\[{\mathrm{G}}]\]
TCGA-AA-3977-01	CRC	R408*	169026061	169026061	C	\[{\mathrm{T}}]\]
TCGA-AA-3947-01	CRC	\[{\mathrm{R}}230{\mathrm{H}}]\]	169021405	169021405	G	A
TCGA-AA-3937-01	CRC	\[{\mathrm{V}}522{\mathrm{M}}]\]	169028523	169028523	G	A
TCGA-AA-AOON-01	CRC	\[{\mathrm{R}}479{\mathrm{Q}}]\]	169028395	169028395	G	A
TCGA-AA-A01P-01	CRC	Q392R	169026014	169026014	A	\[{\mathrm{G}}]\]
TCGA-AG-AO02-01	CRC	E108G	169018215	169018215	A	\[{\mathrm{G}}]\]
TCGA-AG-AOO2-01	CRC	E351D	169025500	169025500	A	C
TCGA-AG-A002-01	CRC	E445G	169028293	169028293	A	\[{\mathrm{G}}]\]
TCGA-CA-6717-01	CRC	E67D	169019093	169018093	A	C
TCGA-CA-6717-01	CRC	E381*	169025990	169025980	G	\[{\mathrm{T}}]\]
TCGA-CA-6717-01	CRC	E381D	169025982	169025982	A	C
TCGA-F5-6814-01	CRC	\[{\mathrm{K}}283{\mathrm{N}}]\]	169021643	169021643	G	\[{\mathrm{T}}]\]
TCGA-AA-3966-01	CRC	R10Q	169015449	169015449	G	\[{\mathrm{A}}]\]
TCGA-AM-5821-01	CRC	H90Y	169018160	169018160	C	\[{\mathrm{T}}]\]
TCGA-CA-5256-01	CRC	Q305*	169023596	169023586	C	T
TCGA-IR-A3LA-01	Cervical cancer	L166V	169020517	169020517	C	\[{\mathrm{G}}]\]
TCGA-IR-A3LH-01	Cervical cancer	L333V	169023670	169023670	C	\[{\mathrm{G}}]\]
TCGA-IR-A3LK-01	Cervical cancer	E445K	169028292	169028292	G	A
TCGA-2W-ABYY-01	Cervical cancer	\[{\mathrm{R}}45{\mathrm{H}}]\]	169015554	169015554	G	A
TCGA-V4-A9EI-01	Ocular melanoma	E36K	169015526	169015526	G	A

The data is sourced from https://www.cbioportal.org/.

Idiopathic pulmonary fibrosis (IPF) is a fatal disease characterized by progressive destructive lung scarring, which is promoted by cellular senescence and is characterized by uncontrolled cell division [51]. An analysis of the common genetic background of IPF and cancer based on the data from the Finland-Finland Study and the UK Biobank, based on two large biobanks, revealed that *SPDL1* is associated with a locus inextricably correlated with IPF [21]. Dhindsa et al. noticed that *SPDL1* was significantly upregulated in the lung tissues of patients with IPF, and the results of the variant level analysis demonstrated a novel correlation between IPF and a rare missense variant of *SPDL1* [52]. Individuals carrying the *SPDL1* allele showed neither statistically significant differences in chromosome length nor in clinical or morphological characteristics compared with the rest of the cohort. These results indicated that *SPDL1* increases the risk of IPF via a distinct mechanism.

## 5 SPDL1 promotes the development of some tumors

*SPDL1* is indispensable for a normal cell cycle, and its deletion in human osteosarcoma cells (U2OS) and primary fibroblasts was found to significantly slow cell migration [18]. In oral squamous carcinoma, high levels of *SPDL1* were reported to accelerate proliferation and platinum resistance in oral squamous carcinoma cells (OSCCs) [53]. Inhibition of *SPDL1* expression in lung cancer reduced the proliferation of cells and increased their sensitivity to paclitaxel, an MT-based chemotherapeutic agent [54]. Analysis of a pancancer database revealed that *SPDL1* expression is elevated in most malignant tumors, including OSCC and esophageal and breast cancers [53]. *SPDL1* is considered a pro-oncogene that expedites tumor cell proliferation, invasion, and metastasis, and is closely associated with the prognosis of tumor patients [10].

It has been hypothesized that the effects of *SPDL1* on tumors may involve enhanced CIN, which accelerates tumor progression, invasion, and metastasis by affecting GIN and driving chromosomal segregation.

Overexpression of *SPDL1* was detected in the tumor tissues of patients with OSCC, and its high expression correlated with the stage, tumor grade, and treatment modality and shortened the survival time of patients. *SPDL1* inhibition is cytotoxic to OSCC cells and strengthens their sensitivity to cisplatin. Liu et al. noted that the expression of *SPDL1* was upregulated in esophageal carcinoma (ESCA) tissues and was related to age, grade, lymph node metastasis, cancer stage, and poor prognosis in patients with ESCA; they also reported that silencing of *SPDL1* impeded the proliferation, migration, and invasion of cells [55]. The expression of *SPDL1* is correlated with biological responses induced by prolonged low-dose ionizing radiation in the treatment of prostate cancer. Radiotherapy triggers the positive regulation of *SPDL1* in inflammatory and survival responses during the cell cycle [56]. Wan et al. used a hybrid model to identify associations between *SPDL1*, the cell cycle, and tumor recurrence in small cell lung cancer [54].

### 5.1 SPDL1 has suppressive effects on some tumors

Given the heterogeneous nature of different tumors, *SPDL1* expression varies across tumors. For the first time, recent studies have proven that the overexpression of hSpindly is a powerful independent prognostic factor that contributes to the better survival of PDAC patients [40]. Recent findings have shown that the expression of *SPDL1* mRNA is significantly associated with poor prognosis. Nonetheless, further research revealed that the overexpression of hSpindly can independently predict improved OS, which is not consistent with previous findings.

*SPDL1* also exhibits a repressive function in CRC. It is located downstream of myocardin-related transcription factor B (*MRTF-B*) and is involved in regulating the growth and survival of patients with CRC; its lower expression is significantly related to reduced survival [57]. A corresponding decrease in *SPDL1* expression was detected after knockdown of the *MRTF-B* gene in CRC tumor cells and increased tumor growth in mouse transplants, indicating a negative regulatory effect of *SPDL1* on the invasion and metastasis of tumor cells and further confirming its suppressive effects in CRC.

The oncogenic effect of *SPDL1* has been suggested to be correlated with the induction of cellular senescence [52]. *SPDL1* may hinder cell proliferation by inducing cellular senescence. However, *SPDL1* was not found to be associated with key senescence-promoting genes despite its positive correlation with Ki-67, TRA2B, KIAA1524, PRC1, Skp2, and other factors. The hypothesis that *SPDL1* inhibits cell proliferation by inducing cellular senescence remains unconfirmed.

### 5.2 SPDL1 increases sensitivity to drugs in some tumors

*SPDL1* reduces drug resistance in tumor cells. After *SPDL1* knockdown, the activity of MTs is inhibited, and they become more sensitive to low doses of paclitaxel owing to the prolongation of mitotic time and an increase in the number of multinucleated cells, reinforcing the cytotoxic activity of paclitaxel and facilitating the death of tumor cells [58]. Currently, the development of drug resistance in tumors is one of the most prominent challenges in cancer therapy [59]. Drugs targeting cell cycle-related factors prevent tumor progression and metastasis by curbing tumor cell proliferation [8,60,61]. Commonly used antitumor drugs influence MT dynamics and SAC, leading to mitotic arrest and death in cancer cells, which normally function as SAC. Controlling SACs may increase the sensitivity of cells to chemotherapeutic drugs. Therefore, chemotherapy should take the SAC status of tumors into consideration; however, no clinical research in this regard has been reported to date. In tumors with impaired SAC, anti-MT drugs may give rise to increased chromosomal misclustering and may exacerbate CIN [62].

Microtubule-targeting agents (MTAs) are widely used to treat many types of tumors and cause mitotic cell cycle arrest and cell death during mitosis. Moreover, MTAs prevent mitosis in cancer cells by activating the SAC, thereby ensuring accurate chromosomal segregation [63]. When SAC is silenced, the cytotoxic activity of MTAs is affected by premature mitotic exit (mitotic slippage). The inhibition of hSpindly mediated by *SPDL1* knockdown results in the mis-segregation of chromosomes and the accumulation of cells in mitosis; these cells become more sensitive to low doses of paclitaxel [58]. This enhanced sensitivity was ascribed to the prolongation of mitotic arrest and an increase in the number of multinucleated cells, both of which are associated with an increase in the cell death response after mitosis.

Therefore, targeting hSpindly by silencing SAC and chromosome attachment can elevate the sensitivity of tumor cells to MTAs, enhance the killing of tumor cells, and reduce the drug resistance of tumor cells.

## 6 Conclusions

hSpindly plays critical roles in cell cycle progression, mitosis, cell cycle checkpoint silencing, MT attachment, and other biological processes that affect cell proliferation and migration. Abnormal expression of *SPDL1* is associated with GIN and promotes the development and progression of many cancers including breast, prostate, and lung cancers. *SPDL1* also increases sensitivity to low-dose MTAs and reduces drug resistance in tumor cells. Interestingly, *SPDL1* exhibits tumor heterogeneity in CRC and PDAC and inhibits the development of these cancers. These studies suggest the diversity of roles played by hSpindly and can help identify new targets and therapeutic strategies for targeting this protein for the treatment of several diseases.
